# Changes of Treg and Th17 cells balance in the development of acute and chronic hepatitis B virus infection

**DOI:** 10.1186/1471-230X-12-43

**Published:** 2012-05-01

**Authors:** Liang Xue-Song, Li Cheng-Zhong, Zhou Ying, Wan Mo-Bin

**Affiliations:** 1 Department of Infectious Diseases, Changhai Hospital, Second Military Medical University, Shanghai, 200433, China; 2 Department of Biochemistry and Molecular Biology, College of Basic Medical Sciences, Second Military Medical University, Shanghai, 200433, China

**Keywords:** HBV, Treg, Th17, Immune

## Abstract

**Background:**

Many studies suggest that in chronic hepatitis B virus (HBV) infection regulate T (Treg) cells and interlukin-17-producing T help cells (Th17) are mutually antagonistic in the immune response. This study is aimed to reveal the cell differentiation environment and the significance of Treg and Th17 balance in the development of acute and chronic HBV infection.

**Methods:**

Ten patients with acute HBV infection (AHB) and forty-eight patients with chronic HBV infection, including 12 asymptomatic HBV carriers (HBV carriers), 18 chronic hepatitis B patients (CHB) and 18 acute-on-chronic HBV-related liver failure (ACHBLF) were enrolled. Treg and Th17 cells differentiation related cytokine levels were detected by using ELISA. Flow cytometry was employed to count the Treg and Th17 frequency in peripheral blood.

**Results:**

Compared to health controls both AHB and ACHBLF patients favoured Th17 cell differentiation, accompanied by a higher proportion of peripheral Th17 cells (P < 0.01) and high level of interleukin-17A (IL-17A) (P < 0.01). However, asymptomatic HBV carriers and CHB were conducive to Treg cell differentiation. In AHB and ACHBLF, peripheral blood IL-17A + CD4 + T cell frequency increased significantly compared with healthy controls. Changes of Treg and Th17 cell frequency were not completely consistent. Both CHB and ACHBLF had lower level of Treg/Th17 ratio than in health control (P < 0.05). Both plasm IL-17A levels (r = −0.72, p<0.001) and Th17 frequency(r = −0.49, p = 0.0003) negatively correlated with plasma HBV DNA load in patients with chronic HBV infection. In addition, both Th17 frequency and plasm IL-17A levels positively correlated with ALT (r = 0.33,p = 0.01 Vs r = 0.29,p = 0.04) and total bilirubin levels (r = 0.72,p<0.0001 Vs r = 0.53,p = 0.0001) in these chronic HBV-infected subjects. However, for AHB there were positive correlation between both Th17 frequency (r = 0.64, p = 0.04) and plasm IL-17A levels (r = 0.69, p = 0.02) with serum ALT levels, but no significant correlation between both HBV DNA level and total bilirubin level with Th17 frequency or plasm IL-17A levels were found. Furthermore, Treg/Th17 ratio was negatively correlated with total bilirubin levels (r = −0.41, p = 0.004) in chronic HBV-infected patients, especially in patients with ACHBLF (r = −0.69,p = 0.001) and positively correlated with viral load in these chronic HBV-infected subjects (r = 0.55, p<0.0001).

**Conclusions:**

Th17 cells are involved in acute and chronic HBV infection, especially in AHB and ACHBLF. CHB and ACHBLF patients manifested obvious Treg/Th17 ratio imbalance, which might be linked to disease progression and the continuous HBV infection.

## Background

Hepatitis B virus (HBV) can cause acute and chronic infection. Chronic infection is closely related with liver cirrhosis and hepatocellular carcinoma [1]. More than 2 billion people have been infected with HBV globally and there are still approximately 350 million chronic HBV infection victims or HBV carriers [2]. HBV do not directly cause liver cell injury. The outcomes after infection are closely related to the host immune response. Appropriate immune response can lead to viral clearance and recovery, excess immune response can lead to liver failure and inadequate immune response will result in sustained HBV infection [3-6].

Foxp3 + CD4 + CD25 + regulatory T cells (Foxp3 + Treg) play an anti-inflammatory role mainly through contact dependent suppression or releasing anti-inflammatory factors on other immune cells such as CD4+ and CD8 + T cells, natural killer (NK) cells and NKT cells, B cells and dendritic cells (DC) [7-9]. Thus, Foxp3 + Treg cells are considered to be of great importance in maintaining self-tolerance, the immune balance and preventing autoimmune diseases, allergies and other immune pathological conditions. In chronic hepatitis B (CHB), Foxp3 + Treg cells are closely related with the development and progress of the disease [10-12].

CD4 + T cells secreting interleukin-17 (IL-17) are a newly established T helper cell subset (Th17), defferent from Th1 and Th2 cells. Th17 cells originate from the same naive cells with Foxp3 + Treg cells, mainly secreting pro-inflammatory cytokines IL-17A, which is linked to inflammation and host antimicrobial immunity [13,14]. Evidence has shown that circulating IL-17 + T cells are largely accumulated in the livers of CHB patients and that their frequency increases with progression from CHB to acute-on-chronic liver failure (ACLF) [15-19].

In general, Foxp3 + Treg and Th17 cells are both involved in the pathogenesis of CHB. Foxp3 + Tregs and Th17 cells are closely associated with each other, to tie Foxp3 + Treg cells with Th17 cells, Zhang JY et al. used the ratio of Foxp3 + Treg cells to Th17 cells as an index and found that Treg/Th17 ratios were decreased in CHB patients compared with HCs, and entecavir-induced suppression of HBV replication lead to a significant reduction of Treg/Th17 ratios [20]. Zhai S et al. also used Treg/Th17 ratios to characterize Th-17 and Treg cells in the blood of HBV-associated ACLF patients and found that the ratio of Th17 to Treg cells is associated with survival of patients with HBV-associated ACLF [21]. However, the relationship of these two types of cells in the development of acute and chronic HBV infection is still not clearly studied. To better illustrate these two closely related immune cells in the development of acute and chronic HBV infection, we detected the frequency of peripheral blood Foxp3 + Treg and Th17 cells and related cell differentiation factors in 58 cases of acute and chronic HBV infection. Compared with the healthy controls, Th17 cells were significantly increased in patients with acute HBV infection (AHB) and acute-on-chronic HBV-related liver failure (ACHBLF), slightly increased in the CHB patients and not changed in asymptomatic HBV carrier. CHB and ACHBLF patients manifested obvious Foxp3 + Treg and Th17 immune imbalance, which was negatively correlated with the liver injury and positively correlated with HBV DNA levels in chronic HBV related subjects.

## Results

### Foxp3 + Treg and Th17 cell differentiation factor levels in the HBV infected patients

**Figure 1:**
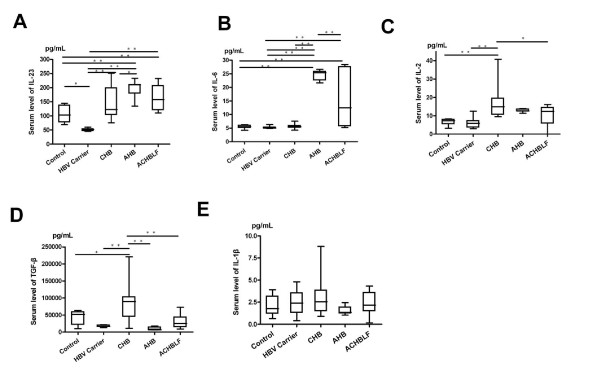
**Serum level of Th17 and Treg-related cytokines in patients with HBV infection. (A)** Interleukin IL-23, **(B)** IL-6, **(C)** IL-2,**(D)** transforming growth factor (TGF)-β1,and **(E)** (IL)-1β were tested. Data are presented as mean ± standard deviation and analyzed by one-way ANOVA analysis. CHB, chronic hepatitis B; NC, normal control, AHB, acute hepatitis B, and ACHBLF, acute-on-chroni HBV-related liver failure. ** P < 0.01;*P < 0.05.

In order to learn about the Foxp3 + Treg and Th17 cells differentiation environment in HBV infected patients, we selected five cytokines primarily related to Foxp3 + Treg and Th17 cell differentiation: interleukin-1beta (IL-1β), interleukin-23(IL-23), interleukin-6(IL-6), transforming growth factor-beta1 (TGF-β1) and interleukin-2 (IL-2). As shown in Figure 1, compared with the healthy controls, the differentiation environment in both AHB and ACHBLF was conducive to Th17 cell differentiation, which was demonstrated by significantly elevated IL-23 and IL-6 levels (Figure 1A, B). In CHB, the environment benefited Foxp3 + Treg cells differentiation, demonstrated by higher IL-2 and TGF-β1 levels (Figure 1C, D). Asymptomatic HBV carrier had similar Foxp3 + Treg and Th17 cells differentiation environment with healthy controls. For all HBV infected patients, the level of IL-1β not changed significantly (Figure 1E).

### Peripheral blood Foxp3 + Treg and Th17 cells levels and their effectors

**Figure 2:**
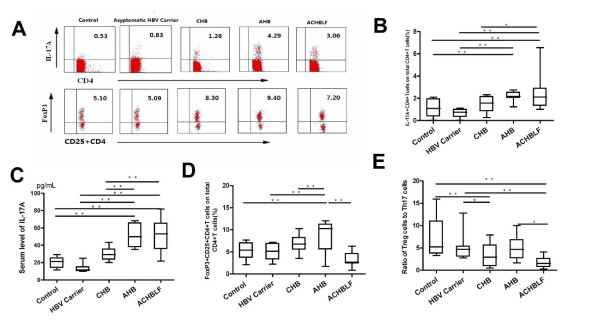
**Distribution of Th17 and Treg cell subset population in each group.(A)** Representative dotplots of intracellular IL-17 and Foxp3 staining in different HBV infected patients; **(B)** Percentage of IL-17A + CD4 + T cells on total CD4 + T cells; **(C)** Serum level of IL-17A; **(D)** Percentage of FoxP3 + CD25 + CD4 + T cells on total CD4 + T cells; **(E)** Ratio of Treg cells to Th17 cells. CHB, chronic hepatitis B; AHB, acute hepatitis B, and ACHBLF, acute-on-chronic HBV-related liver failure.** P < 0.01;*P < 0.05.

Based on the knowledge of Foxp3 + Treg and Th17 cells differentiation factors in acute and chronic HBV infection, we further evaluated the Foxp3 + Treg and Th17 cells level in different stages of HBV infection in peripheral blood, and the results were shown in Figure 2. In both AHB and ACHBLF patients, peripheral blood IL-17A + CD4 + T cell frequency increased significantly compared with the healthy controls.(AHB:2.18 ± 0.42%;ACHBLF:2.38 ± 1.32%; Control:1.07 ± 0.23%) (Figure 2B). Peripheral blood IL-17A level consistent with Th17 cell frequency in AHB and ACHBLF, and the IL-17A level was significantly higher than that in healthy controls and asymptomatic HBV carriers or CHB (Figure 2C). Despite Foxp3 + Treg cells generate from the same naive T cell poll that generates Th17 cells, we found changes of the frequency of Foxp3 + Treg cells and Th17 cells were not completely synchronous during the development of acute and chronic HBV infection except in AHB stage, increased significantly accordingly (AHB, 8.83 ± 3.65% Vs healthy controls 5.30 ± 1.82%, P <0.001)(Figure 2D).

Given the nonsynchronous changes of Foxp3 + Treg and Th17 cells in different stages of HBV infection, to better correlate these two types of immune cells closely in functions and differentiation, we used Foxp3 + Treg/Th17 ratio to describe their relationship. The results showed that compared with the control group (5.41 ± 1.21), Treg/Th17 ratios in ACHBLF group (1.74 ± 0.25, P <0.001) and CHB group (3.25 ± 0.63, P <0.05) were significantly decreased and the ratios in the AHB group (4.97 ± 0.78, P > 0.05) and HBV carrier group (6.29 ± 0.94, P > 0.05) did not change significantly (Figure 2E).

### Relationship between Th17 population and liver injury in acute and chroinc HBV infection

**Figure 3:**
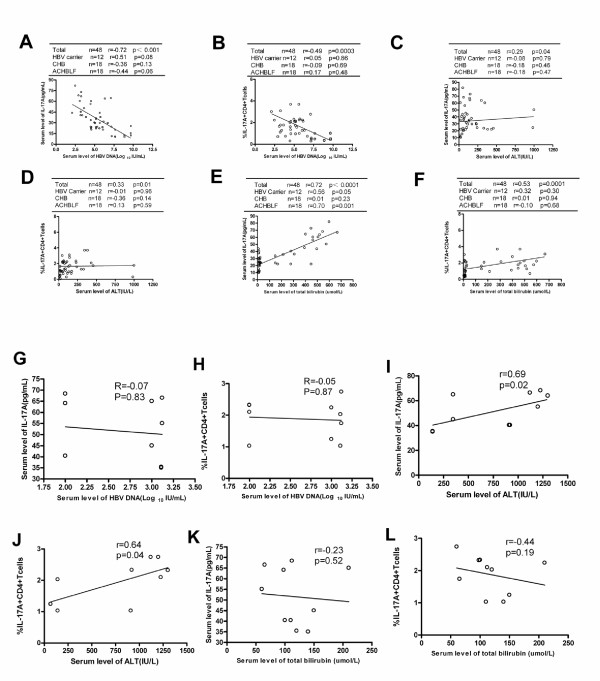
**The relationship between peripheral Th17 frequency/serum IL-17A level and virological/biochemical characters of patients with HBV infection.** Peripheral Th17 frequency or serum IL-17A level negatively correlated with plasma HBV loads **(A,B)** and positively correlated with serum ALT or total bilirubin level **(C,D,E,F)** in total chronic HBV infected subjects. In AHB, Th17 frequency and serum IL-17A level only positively correlated with ALT **(I, J)**, but not significantly correlated with HBV DNA level and total bilirubin level **(G,H,K,L)**. Solid line, linear growth trend; r, correlation coefficient. P-values are shown.

To further detect the relationship between the Th17 cells frequency and liver injury, we analyzed correlation between either Th17 frequency or its main effector IL-17A and plasma HBV DNA load or ALT levels and total bilirubin levels in these acute and chronic HBV infected patients. For chronic HBV infected patients, there were some significant negative correlations between plasma HBV DNA load and both plasma IL-17A levels (r = −0.72,p<0.001; Figure 3A) and Th17 frequency (r = −0.49,p = 0.0003; Figure 3B). Further analysis indicated that these negative association did not occur in different separate chronic HBV infected cohorts (Figure 3A, B). In addition, we found that both Th17 frequency and plasma IL-17A levels positively correlated with ALT and total bilirubin levels (Figure 3C, D, E, F) in these chronic HBV-infected subjects, but further analysis found that these positive association existed only between plasma IL-17A levels and total bilirubin levels in patients with ACHBLF(r = 0.70,p = 0.001) (Figure 3F). However, there were positive correlation between both Th17 frequency(r = 0.64,p = 0.04) and plasma IL-17A levels with serum ALT levels(r = 0.69,p = 0.02), but no significant correlation between both HBV DNA level and total bilirubin level with Th17 frequency or plasma IL-17A levels were found in AHB(Figure 3G-L). No clear correlation between Foxp3 + Treg cell frequency and liver injury was detected.

**Figure 4:**
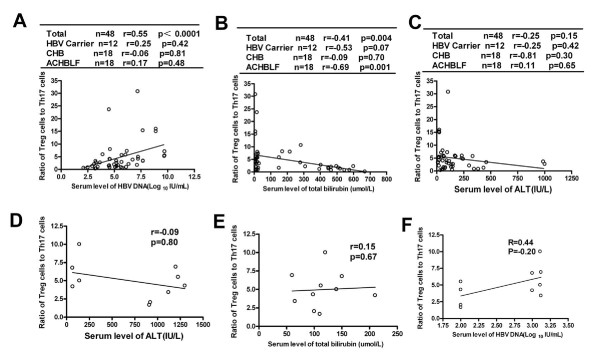
**The ratio of Treg cells to Th17 cells negatively correlated with total bilirubin levels in chronic HBV-infected patients, especially in patients with ACHBLF (B) and positively correlated with viral load in these chronic HBV-infected subjects (A).** No significant correlation between Treg/Th17 ratio and serum ALT levels in these chronic HBV infected subjects were found **(C)**. Furthermore, there were no significant correlation between Treg/Th17 ratio and liver injury or viral load in AHB **(D, E, F)**.

Further analysis of Treg/Th17 ratio with the degree of liver injury and viral load showed that Treg/Th17 ratio positively correlated with viral load in these chronic HBV-infected subjects (r = 0.55, P<0.0001) (Figure 4A) and negatively correlated with total bilirubin levels (r = −0.41, P =0.004) in chronic HBV-infected patients, especially in patients with ACHBLF (r = −0.69, p = 0.001) (Figure 4B). There was no significant correlation between Treg/Th17 ratio and serum ALT levels in patients with chronic HBV infection (Figure 4C). Furthermore, there were no significant correlation between Treg/Th17 ratio and liver injury or viral load in AHB (Figure 4D, E, F).

## Discussion

Immune-mediated liver injury is an important pathogenesis of HBV infection. Recent studies focused on the roles of Treg cells in persistent HBV infection [10-12]. Newly discovered Th17 cells shared the same naive T cells with Treg cells and the same signaling pathways of cell differentiation. Considerable development plasticity has attracted great attention [22-24]. In this study, Treg and Th17 cell frequency and related differentiation cytokine environment in acute and chronic HBV infected patients were detected. We found that in AHB and ACHBLF, peripheral blood Th17 cell percentage of the total CD4+ T cells increased significantly compared with the healthy controls. We found no significant difference between the healthy controls and asymptomatic HBV carriers or CHB. In addition, change of peripheral blood IL-17A levels consistent with Th17 cells and increased in AHB and ACHBLF. Besides, peripheral IL-17A level and Th17 cell frequency positively correlated with liver injury in AHB or chronic HBV infected subjects. For AHB IL-17A level and Th17 frequency positively correlated with ALT level and for chronic HBV-infected subjects especially ACHBLF they were positively correlated with total bilirubin level. Ge et al. [18] found that peripheral blood Th17 cell levels in active chronic HBV infection were significantly higher than those in the healthy controls associated with elevated serum levels of IL-17A. And Ye et al. [17] and Wang et al. [25] demonstrated that IL-17 secretion cells in the liver of CHB and peripheral Th17 cells increased among patients with CHB compared with healthy controls associated with fibrosis and inflammation degrees. All these findings strongly suggest that Th17 cells participate in liver injury process and viral clearance after HBV infection. Furthermore,we found that both Th17 frequency and plasma IL-17A levels were reversely correlated with viral load in total chronic HBV-infected subjects. These results were in contrast to the findings of Zhang et al.[15]. The difference may be due to the different study groups and detection methods used.

Differentiation processes of Th17 and Treg cells cross with each other [26-28]. In the mouse model, studies found that inflammatory cytokines IL-6 could completely block the FoxP3 + Treg cells differentiation and IL-6 with TGF-β could induce Th17 cell differentiation [22]. In view of the connection between Treg and Th17 cells, we examined the Treg and Th17 cells differentiation microenvironment in different stages of HBV infection and found that IL-23 and IL-6 levels in AHB were significantly higher than those in chronic HBV infection and the healthy controls. On the contrary, TGF-β and IL-2 levels were significantly elevated in patients with CHB. All these results suggest that change of Th17 and Treg cell level mainly originate from the host immune micro-environment change due to HBV infection. Recently Zhang et al. [29] also obtained similar results and demonstrated that in vitro blocking IL-6 and IL-6 receptors combination could reverse IL-17 secretion cells differentiation. These results suggest that in the future we can adjust the IL-6/IL-6R pathway to regulate the immune process of HBV infection so as to achieve the purpose of immunotherapy.

Th17 cells and Treg cells not only share the same origin but also are mutually antagonistic in function. Thus, the balance between the two could impact the inflammation control and autoimmune inflammation [28]. Many studies have found that imbalance between Th17 and Treg cells is closely related to the development of a number of diseases [24,30-32]. Wang et al. [25] examined the Foxp3 expression and IL-23/IL-17 cytokines gene expression levels of cells infiltrating the liver of the HBV infected and found that the two were all increased compared with the healthy controls. But they did not study further the correlation between the two and disease progression. In this study, we applied Treg/Th17 ratio to express the relationship between the Foxp3 + Treg cells and the Th17 cells and demonstrated its changes in the HBV infection process. We found that compared with the healthy controls, peripheral blood Treg/Th17 ratio declined in CHB and ACHBLF and did not significantly change in AHB and HBV carriers. Therefore, we concluded that Treg and Th17 immune imbalance existed in CHB and ACHBLF stages. In addition, we found that for total chronic HBV-infected patients,Treg/Th17 ratio positively correlated with peripheral viral load and reversely correlated with total bilirubin level especially in ACHBLF. Zhang JY et al. [20] also found Treg/Th17 ratios were decreased in CHB patients compared with HCs, and antiviral therapy induced suppression of HBV replication lead to a significant reduction of Treg/Th17 ratios. These data indicated the imbalance of Treg cells to Th17 cells might play an important role in HBV persistence.

## Conclusion

The results suggest that IL-17 + CD4 + T cells participate in the inflammatory process of acute HBV infection and chronic HBV infected subjects especially patients with ACHBLF. Foxp3 + Treg and Th17 immune imbalance exists in CHB and ACHBLF. Immunosuppression by Treg cells mainly occurs in CHB and Th17-mediated inflammatory damage mainly occurs in ACHBLF. In this study, we only detected peripheral blood Th17 cells and Treg cells in HBV infected patients and did not further study the interaction of these two cells in vitro. Nor did we follow up the CHB and ACHBLF patients dynamically. Thus we could not learn about the exact underlying mechanisms, which still need further study.

## Methods

### Patients

A total of 58 patients with HBV infection [44 men and 14 women; median age 38 years (range: 21 ~ 68 years)] were included in the present study. Patients were classified into the following 4 groups. (1) the asymptomatic HBV carriers (belonged to immune tolerance phase) [8 men and 4 women, median age 25 years (range: 22 ~ 34 years)]: positive for hepatitis B surface antigen (HBsAg) and hepatitis B core antigen (anti-HBc), negative for antibodies (Abs) to HCV, delta virus (HDV), hepatitis G virus (HGV), HIV-1 and −2, without other causes of chronic liver damage, with the presence of HBeAg, high serum HBV DNA level, normal ALT, and minimal or no evident inflammation in the liver biopsy. (2) AHB patients [(8 men and 2 women, median age 40 years (range:32 ~ 45 years)]: with high serum titer of HBsAg and antibody to anti-HBc of the immunoglobulin M (IgM) and acute-onset elevation of serum ALT levels, excluding other origins of acute hepatitis, and with confirmed absence of HBsAg 6 months before admission (They all had negative results for serum HBsAg test). (3) CHB patients (belonged to immune clearance phase or low HBV DNA replication phase) [14 men and 4 women, median age 37 years (range:21 ~ 68 years)]: positive for HBsAg and anti-HBc for more than 6 months, negative for Abs to HCV, HDV, HGV, and HIV-1 and −2, and without other causes of chronic liver damages, and with persistently elevated serum ALT level and positive serum HBV DNA. (4) ACHBLF patients [14 men and 4 women, median age 45 years (range:30-65 years)]: (a) HBV-related liver cirrhosis based on a histopathologic diagnosis or compatible laboratory data and sonographic findings, (b) recent development of jaundice, ascites, hemodynamic instability and/or encephalopathy grade III–IV compatible with the definition of hepatic decompensation necessitating further treatment in the ICU, (c) no evidence of hepatocellular carcinoma or other metastatic liver tumour that can affect liver function, and (d) no immunosuppressive medication within the least 3 months prior to study entry. None of these patients had concurrent HCV, hepatitis G virus, and HIV infections or autoimmune liver diseases. The blood of patients with AHB and ACHBLF were collected at the time of peak of total bilirubin and all the blood of patients with CHB were collected at the time of diagnosis and before receiving antiviral therapy. The clinical and biochemical details of the patients were listed in Table 1. Ten healthy blood donors were selected as normal control. All patients and controls were Chinese. This study was approved by the Ethics Committee of Changhai Hospital. Written informed consent was obtained from each patient.

**Table 1:** Clinical details of the studied patients

	**Normal control**	**HBV carrier**	**AHB**	**CHB**	**ACHBLF**
**Number**	10	12	10	18	18
**Age (years)**	30 (24 ~ 42)	25(22 ~ 34)	40 (32 ~ 45)	37 (21 ~ 68)	45 (30 ~ 65)
**Gender(male/female)**	7/3	8/4	8/2	14/4	14/4
**HBeAg positive**	NA	12	2	11	7
**HBV DNA**					
**(Log**_**10**_**IU/mL)**	NA	8.3(7.24‒9.72)	3.00(2‒3.12)	5.15(2.12‒6.73)	4.53(2.94‒7.14)
**ALT(IU/L)**	NA	23.5(13‒42)	915(67‒1226)	126(65‒988)	125(21‒1002)
**TBil (μmol/L)**	NA	10.5(5‒16)	111(60‒210)	18.8(8.9‒151)	457.2(170‒599)
**Encephalopathy grade III/IV(%)**	NA	0	0	0	35.29
**Ascites(%)**	NA	0	0	0	82.35
**Mortality (%)**	NA	0	0	0	23.53

Data are expressed as median (range) or number. NA, not applicable; AHB, Acute hepatitis B; CHB, chronic hepatitis B; ACHBLF, Acute-on-chronic HBV-related liver failure; ALT, Alanine aminotransferase; TBil, total bilirubin; Normal values:ALT,0–60 U/l; total bilirubin, 2–17 μmol/L.

### Flow cytometric analysis

For Th17 cell examination, the PBMCs were isolated from peripheral blood. PBMCs, 2 × 10^6^, were stimulated further for 5 h with 50 ng/ml phorbol myristate acetate, 1 mM ionomycin (both from Sigma, St Louis, MO, USA) and 10 mg/ml brefeldin A (TocrisCookson, Bristol, UK) in complete RPMI-1640 (Invitrogen, Carlsbad, CA, USA) supplemented with 10% heat-inactivated fetal bovine serum (Gibco, Grand Island, NY, USA). Upon harvest, cells were firstly surface-stained with fluorescein isothiocyanate (FITC)-conjugated anti-human CD4 antibodies for 20 min, fixed and permeabilized with Perm/Fixsolution, and then stained intracellularly with phycoerythrin (PE)-conjugated anti-human IL-17A.

For Treg cell examination, peripheral blood (100 μL) was firstly surface-stained with FITC-conjugated anti-human CD4 antibodies and allophycocyanin (APC)-conjugated anti-human CD25 antibodies for 30 min, then lysed with FACSTM lysing solution (BD PharMingen), and treated with eBioscience fix/perm mixture (eBiosciences) according to the manufacturer’s instructions. Finally, the cells were incubated with PE-conjugated anti-human Foxp3 antibodies overnight. Isotope controls were used to ensure antibody specificity. Flow cytometry was performed using FACSCalibur (Becton Dickinson, San Jose, CA). FACS data were analyzed using CellQuest software (Becton Dickinson Rutherford, NJ) [28]. All antibodies were purchased from BD Biosciences (San Jose, CA, USA).

### Enzyme linked immunosorbent assay (ELISA)

Serum concentrations of IL-1β, IL-6, IL-23, IL-17A, TGF-β1, and IL-2 were measured by commercially available ELISA Kits (R&D Systems, Minneapolis, MN, USA) according to the protocols provided by the manufacturer. All samples were assessed in triplicate.

### Virological and biochemical assessments

The levels of HBsAg, HBeAg, anti-HBs, anti-HBc, anti-HBe, anti-HCV, anti-HDV, anti-HGV, anti-HIV-1, and anti-HIV-2 were measured using commercially available kits (Abbot Laboratories, North Chicago, IL) in our clinical lab. Serum HBV-DNA level was measured by fluorescent quantitative PCR with commercially available kits (PE/B/MJ/L, Shenzhen, China) according to the manufacturer’s instructions. The threshold of the HBV DNA detection limit was 500 IU/ml.

### Statistical analysis

Statistical analysis was performed using GraphPad Prism4 software (GraphPad, NJ.U.S.A). Comparison between various individuals was performed using one-way ANOVA. The correlations between the variables were evaluated using the Spearman rank correlation test. For all tests, a P value less than 0.05 was considered statistically significant.

## Abbreviations

HBV, Hepatitis B virus; Foxp3+Treg, Foxp3+CD4+CD25+ regulatory T cell; Th17, Interleukin-17 producing T help cell; CHB, Chronic hepatitis B; AHB, Acute hepatitis B; ACHBLF, Acute-on-chronic HBV-related liver failure; IL-7, Interleukin-17; IL-1β, Interleukin-1 beta; IL-23, Interleukin-23; IL-6, Interleukin-6; TGF-β, Transforming growth factor-beta; IL-2, Interleukin-2; ALT, Alanine aminotransferase.

## Competing interests

The authors declare that they have no competing interests.

## Authors’ contributions

LX-S and LC-Z contributed equally to this work. LX-S conceived of the study, and participated in the design of the study,performed the statistical analysis and drafted the manuscript; L C-Z collected all human materials and helped to draft the manuscript. ZY performed most of the experiments. WM-B conceived of the study, and participated in its design and coordination. All authors read and approved the final manuscript.

## Pre-publication history

The pre-publication history for this paper can be accessed here:

http://www.biomedcentral.com/1471-230X/12/43/prepub
